# Two K2P Channels, TWK-46 and TWK-26 do not affect *C. elegans* Egg-Laying Behavior

**DOI:** 10.17912/micropub.biology.001477

**Published:** 2025-01-27

**Authors:** Li Chen, Isabel Beets, William Schafer

**Affiliations:** 1 Biology, KU Leuven, Leuven, Flanders, Belgium; 2 Neurobiology, MRC Laboratory of Molecular Biology, Cambridge, England, United Kingdom

## Abstract

Two-pore domain potassium channels, also known as K2P channels, play vital roles in maintaining the resting membrane potential in excitable cells, affecting a variety of physiological processes across species. The *Caenorhabditis elegans* ( *C. elegans* ) genome contains 46 different K2P-encoding genes, yet most of their functions remain unknown. Here, we have investigated the possible roles of two *C. elegans* K2P channel genes – *twk-26* and *twk-46* – that are expressed in the egg-laying neural circuit by characterizing the egg-laying behavior of null mutants generated by CRISPR/Cas9 gene editing. However, using a variety of assays, we did not observe significant differences in egg-laying behavior between *twk-26* and *twk-46* mutants and wild-type worms *.*

**Figure 1. Characterization of egg-laying behavior in two C. elegans K2P channel mutants:**
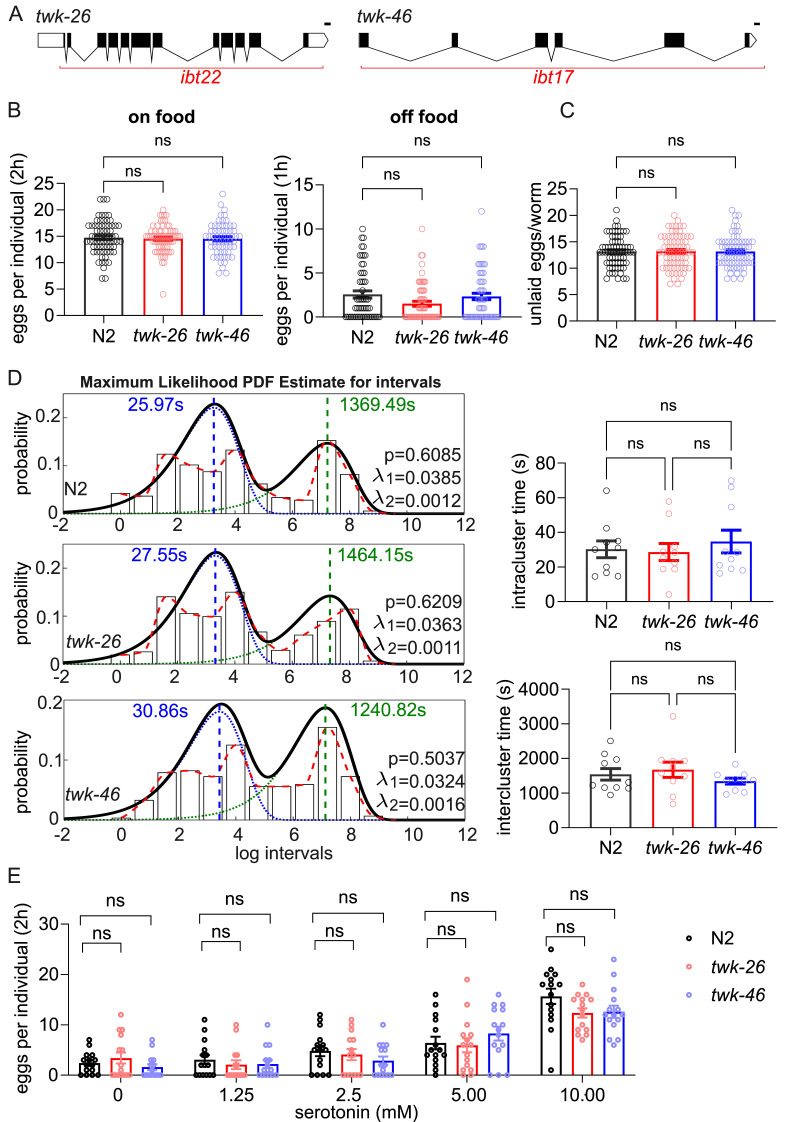
A. Graphic representation of CRISPR full knockout alleles of *twk-26(ibt22)* and *twk-46(ibt17)* (scale bar: 100bp). B. Quantification of the number of eggs laid on food within 2 hours (N2: n=69; *twk-26* : n=70; *twk-46* : n=61 animals) and off food within 1 hour (N2: n=55; *twk-26* : n=63; *twk-46* : n=62 animals). Statistical significance was evaluated using a one-way ANOVA test, ns: p>0.05. C. Quantification of the number of unlaid eggs within uterus (N2: n=72; *twk-26* : n=71; *twk-46* : n=70 animals). Statistical significance (one-way ANOVA test), ns: p>0.05. D. Quantitative analysis of the timing of egg-laying events. Individual animals were tracked on food for 6 hours, and the intervals between egg-laying events were measured. The left panel represents the histogram of log intervals of 10 individual animals of each genotype, with the maximum likelihood estimation (Waggoner et al., 1998; Zhou et al, 1998) of intra- and inter-cluster time constants as well as three parameters of the two-state egg-laying model (p: probability of egg-laying events during active egg-laying phase, *λ1* : rate constant of inactive egg-laying phase, and *λ* 2: rate constant of active egg-laying phase). The blue and green dashed lines indicate the intra- and inter-cluster time constants, respectively. The right panel plots the inter- and inter-cluster time constants determined by the same method per individual animal. Estimated parameters were not statistically different between genotypes according to Brown-Forsythe and Welch ANOVA, with Dunnett's post-hoc test. E. Quantification of the number of laid eggs in response to different exogenous serotonin concentrations within 1 hour in the absence of food (n=15 animals for each genotype). Significance was tested using an Aligned Ranked two-way ANOVA test, ns: p>0.05. For all graphs, error bars represent standard error of the mean (SEM).

## Description

Two-pore domain potassium channels are a class of 4-transmembrane spanning K ^+^ channels, with two pore-forming domains in each subunit (Natale et al., 2021) . In mammals, some K2P genes have been discovered as novel anesthesia targets due to their activation by certain anesthetics and broad expression in the central nervous system (Steinberg et al., 2015; Talley et al., 2001) . Surprisingly, the most diversified K2P gene family has been found in the model organism *C. elegans* (Hobert, 2013) , which has a very compact nervous system with only 302 neurons in hermaphrodites (White et al., 1986) . Different studies have indicated that K2P genes are involved in regulating various activities in *C. elegans* . For instance, *twk-40* impacts both defecation and locomotion by regulating the excitability of the defecation neuron DVB and the premotor neuron AVA, respectively (Meng et al., 2024; Yue et al., 2024) . Likewise, a gain-of-function mutant of *twk-18* exhibits temperature-dependent locomotion defects (Kunkel et al., 2000) . Recently, an unconventional K2P channel *unc-58* has been discovered to be the first Na ^+^ permeable K2P channel in *C. elegans* ; this channel is involved in regulating cellular excitability of both body wall muscles and mechanosensory neuron ALM (Andrini et al., 2024) . Nevertheless *,* the majority of *C. elegans* K2P gene functions are unknown. In particular, with few exceptions such as *twk-40* , most neuronally-expressed K2P channels in *C. elegans* have no described loss-of-function phenotype.

We were interested in investigating the roles of uncharacterized K2P channels in the egg-laying circuit. The egg-laying motor program is carried out by a simple circuit, with 12 pairs of vulval muscles innervated by two types of hermaphrodite-specific neurons: the HSNs and VCs (White et al., 1986) . The HSN neurons release serotonin, acetylcholine and the neuropeptide *nlp-3* to switch worms from an inactive phase to an active phase in which eggs are released (Brewer et al., 2019; Waggoner et al., 1998) . The two vulval-proximal VC neurons, VC4 and VC5, can also promote vulval muscle activity via the release of neurotransmitters (Kopchock et al., 2021; Waggoner et al., 1998) . Both these neural types express a range of potassium channels that regulate their membrane potential, and gain-of-function mutations in several have been shown to affect egg-laying behavior. For example, gain-of-function alleles of *egl-36* (a voltage-gated K channel) and *egl-23* (a K2P channel) show severe egg retention in the uterus, possibly due to the hyperpolarization and inactivity of the vulval muscles (Ben Soussia et al., 2019; Elkes et al., 1997; Reiner et al., 1995) . Single-cell RNA sequencing data have identified more K2P channels whose expression is enriched in HSN and VC neurons (Taylor et al., 2021) . The effects of loss-of-function mutations in these K2P channels expressed in egg-laying neurons has not been investigated.

To explore whether K2P channels affect egg-laying behaviors via HSN, VC4 and VC5 neurons, we explored the expression data of all K2P genes enriched in these neurons using the CenGEN app (threshold 2, filtered by TPM value > 10, Taylor et al., 2021). This analysis revealed 7 K2P genes showing clear expression in the VC4 and VC5 neurons and 3 K2P genes expressed in the HSNs. Among them, *twk-26* and *twk-46* were selected for further studies due to their high expression in both neuron classes. We generated a null allele for *twk-26* ( *ibt22* ) and *twk-46* ( *ibt17* ) using CRISPR/Cas9 gene editing ( Figure 1A ).

To determine if either of these mutants has egg-laying phenotypes, we carried out a range of egg-laying assays for both mutants. We first compared egg-laying rates of wild-type and mutant worms, under both on-food and off-food conditions. Under the on-food condition, *twk-26* and *twk-46* mutants showed no significant differences in eggs laid on food compared to wild-type worms ( Figure 1B, left panel). Food is an important environmental factor influencing egg-laying behaviors, and the absence of food can inhibit egg-laying events (Trent C, 1982) . We therefore investigated whether mutants lacking *twk-26* or *twk-46* might lay more eggs in the absence of food due to possibly reduced hyperpolarization of the egg-laying motor neurons. However, under the off-food condition, *twk-26* and *twk-46* mutants showed no differences compared to wild-type in the number of eggs laid within 1 hour ( Figure 1B, right panel). In addition, neither of the two mutants showed a significant difference in the number of eggs retained in the uterus compared to wild-type worms ( Figure 1C ).

We also investigated whether the temporal egg-laying pattern on food was altered in the *twk-26* and *twk-46* mutants. In *C. elegans* , animals fluctuate between an inactive phase, in which eggs are retained, and an active phase, in which eggs are laid in clusters. Both the onset of the active phase and egg-laying within the active phase are Poisson random processes with characteristic time constants (Waggoner et al., 1998; Zhou et al 1998) . We made 6-hour recordings of wild-type and mutant worms, and compared their egg-laying patterns by estimating their inter- and intra-cluster egg-laying time constants. We found that the *twk-26* and *twk-46* mutant laid eggs in a clustered pattern that resembled that of wild-type ( Figure 1D, left panel). Furthermore, by comparing the intracluster and the intercluster egg-laying time intervals per individual animal, we observed that the temporal pattern of egg-laying in *twk-26* and *twk-46* null mutants was not significantly different from wild-type ( Figure 1D, right panel).

We also investigated the effect of *twk-26* and *twk-46* on egg-laying behavior evoked by serotonin. Exogenous serotonin can induce egg-laying in wild-type, as serotonin is one of the main neurotransmitters that elicit active phase initiation and vulval muscle contraction (Horvitz et al., 1982; Shyn et al., 2003) . We hypothesized that these two K2P mutants might respond differently to a range of concentrations of exogenous serotonin due to the possibly less-hyperpolarized HSN neurons. However, over a range of exogenous serotonin concentrations from 0 mM to 10 mM, *twk-26* and *twk-46* mutants showed no significant differences in the number of eggs laid compared to wild-type, suggesting that their sensitivity to serotonin is not altered ( Figure 1E ).

To conclude, we observed no abnormalities in egg-laying behavior in CRISPR/Cas9 full knockout single mutants of *twk-26* and *twk-46* , despite carrying out a series of sensitive assays including egg retention, egg-laying rate on and off-food, egg-laying temporal pattern and sensitivity to exogenous serotonin. This may indicate significant redundancy between these and other K2P channels expressed in egg-laying neurons. Alternatively, *twk-26* and *twk-46* -encoded channels may conduct only minor potassium currents, which are potentially too small to cause behavioral changes in loss-of-function mutants. Further experiments using calcium imaging of HSN, VC4 and VC5 neurons in these two mutants could shed light on the *in vivo* function of *twk-26* and *twk-46* at the neuronal level.

## Methods

*C. elegans* strains and maintenance

**Table:** 

Strain name	genotype	source
N2	*C. elegans* wild isolate	*Caenorhabditis* Genetics Center (CGC)
IBE560	*twk-46 ( ibt17 ) V *	this study
IBE622	*twk-26 ( ibt22 ) X *	this study

All strains were maintained on NGM (nematode growth medium) plates seeded with *E. coli* strain OP50 in a 20°C incubator.

CRISPR/Cas 9 gene editing

Selection of PAM sites and crRNA design were done via http://crispor.gi.ucsc.edu/ , where the least off-target and mismatch option was selected. Repair templates contain 70 bp nucleotides with 35 bp flanking two PAM sites. Sequences of crRNAs and repair templates used for generating *twk-26* and *twk-46* deletion mutants are included in the table below.

**Table:** 

knockout mutant	crRNAs	repair template
*twk-26 ( ibt22 ) *	crRNA1: ATTGCCTCCCGTTTCTACAG	cacttttccgttcaacgcgggttcggagacaaaagttcagtttgatctacgaagatctacaaaatcgcgg
	crRNA2: ATGGGACACTGACACCATGT	
*twk-46 ( ibt17 ) *	crRNA1: AAAAAGATGAGAACAAAATT	aaaaaaatttttttttggttttttttcgcaaacttaattaaaaaaacgataatccgcaagattcataaaa
	crRNA2: GTGAATAACAATATTCGATA	

CRISPR injection mixtures for *twk-26* and *twk-46* gene edits were made as previously described in Dokshin et al., 2018, using a *dpy-10* co-CRISPR strategy. In the F1 progeny, rollers were picked and genotyped for heterozygous *twk-* 26 or *twk-46* mutations. From the heterozygous F1 progeny, non-Dpy F2 progeny were picked and genotyped to identify homozygous mutants. Homozygous null mutants were sequence-verified by Sanger sequencing. Endpoints for both alleles are included in the table below.

**Table:** 

allele	description
*ibt17*	*twk-46* full gene deletion allele; a 6966 bp fragment covering the whole *twk-46* gDNA including 5' and 3' UTR was deleted; endpoints of deletions: aaaaaaatttttttttggttttttttcgcaaactt//aattaaaaaaacgataatccgcaagat
*ibt22*	*twk-26* full gene deletion allele; a 4220 bp fragment covering the whole *twk-26* gDNA including partial 5' UTR and full 3' UTR was deleted; endpoints of deletions: gttcggagacaaaagattgcctcccgtttcta// GGCTA //ggtgtcagtgtcccatttcagtttgatcta ( GGCTA is an unexpected indel inserted during CRISPR editing)

Worm synchronization

Around 18 hours before the assay, worms were synchronized by picking late L4 stage worms with a clear half circle structure and blinded. Day-1 adults were used for all assays.

Egg-laying assays on/off food

For the on-food condition, small plates (35 mm) were poured with NGM and seeded with 10 μL of an overnight OP50 culture. Individual synchronized day-1 worms were transferred to the bacterial patch of a single plate. After 2 hours, worms were removed and the eggs on each plate were counted. Three independent assays were conducted on three days; each replicate had at least 20 individual worms for each genotype. For the off-food condition, 24-well flat-bottom plates filled with NGM were prepared beforehand. Worms picked from their culture plate were first allowed to crawl on an empty NGM plate for a few minutes to remove bacteria. Individual bacteria-free worms were transferred to a well of the 24-well plates. After 1 hour, worms were removed and the eggs in each well were counted. Four independent assays were conducted on four days; each replicate had at least 12 individual worms for each genotype.

Egg-laying tracking assay

Individual synchronized day-1 worms were placed on an NGM plate (55 mm) seeded with 10 μL OP50 culture. 6-hour videos were recorded for each plate using an in-house 6-camera (Dinolite) setup. Egg-laying timepoints were manually marked using the software Kinovea v.0.9.5 and exported for further analysis. The histogram of log intervals and three interval-related parameters p, *λ* 1, and *λ* 2 were obtained using a MATLAB (R2002a) script adapted from Waggoner et al., 1998 and Zhou et al., 1998. For each individual, the intercluster interval was determined by 1/ *λ* 1, and the intracluster interval was calculated as 1/p* *λ* 2. For each genotype, a total of 10 animals were recorded for 6h on 5 different days.

Egg retention assays

Synchronized day-1 adult worms were placed between two cover slides with 3 μL S basal (5.85 g NaCl, 6 g KH _2_ PO _4_ , 1 g K _2_ HPO _4_ , filled up to 1 L with Milli-Q ultrapure water, autoclaved) and 3μL 1M NaN _3_ . Eggs in the uterus of each animal were counted using a Zeiss Axio Observer inverted microscope with 20x objective. Three independent assays were conducted on three days; each replicate has at least 20 individual worms for each genotype.

Exogenous serotonin assays

Different concentrations of serotonin creatinine sulfate monohydrate in M9 buffer (6 g Na _2_ HPO _4_ , 3 g KH _2_ PO _4_ , 5 g NaCl, filled up to 1 L with Milli-Q water, after autoclaving, 1 mL 1 M MgSO _4_ was added) were made freshly on the day of the assay and pipetted into a 96-well flat-bottom plate (100 μL/well). Worms picked from their culture plate were first allowed to crawl on an empty NGM plate for a few minutes to remove bacteria. Then, individual worms were placed in a well containing exogenous serotonin. After 2 hours, eggs were counted in each well for further analysis. Three independent assays were conducted on three days; each replicate had 5 individual worms for each concentration and each genotype.

Statistical analysis

For the egg-laying assay on food, a one-way ANOVA with Dunnett's post-hoc test was performed using GraphPad Prism v10.2.2. For the egg-laying assay under the off-food condition, a Brown-Forsythe and Welch ANOVA with Games-Howell post-hoc test was performed using GraphPad Prism v10.2.2. For the egg-laying tracking assay, a Brown-Forsythe and Welch ANOVA with Dunnett's post-hoc test was performed with GraphPad Prism v10.2.2. For the exogenous serotonin assays, an Aligned Ranked ANOVA with Holm's post-hoc test was performed in R studio v2024.12.0.

## Data Availability

Description: Matlab script for egg-laying parameter estimation. Resource Type: Software. DOI: https://doi.org/10.22002/86a6s-k7y43
